# Probing the evaporation dynamics of semi-volatile organic compounds to reveal the thermodynamics of liquid–liquid phase separated aerosol†

**DOI:** 10.1039/d3sc05164a

**Published:** 2024-01-17

**Authors:** Jack M. Choczynski, Bilal Shokoor, Jorge Salazar, Andreas Zuend, James F. Davies

**Affiliations:** a Department of Chemistry, University of California Riverside Riverside CA USA jfdavies@ucr.edu; b Department of Atmospheric and Oceanic Sciences, McGill University Montreal Quebec Canada

## Abstract

Liquid–liquid phase separation (LLPS) is a thermodynamically driven process that occurs in mixtures of low miscibility material. LLPS is an important process in chemical, biological, and environmental systems. In atmospheric chemistry, LLPS in aerosol containing internally-mixed organic and inorganic particles has been an area of significant interest, with particles separating to form organic-rich and aqueous phases on dehydration. This alters the optical properties of the particles, has been connected to changes in the cloud nucleation ability of the aerosol, and potentially changes the reactivity of particles towards gas-phase oxidants. Although the chemical systems that undergo LLPS have become quite well-characterized, the properties and processes of LLPS particles are quite poorly understood. In this work, we characterize LLPS in aerosol particles containing ammonium sulfate and triethylene glycol (3EG), a semi-volatile organic molecule. We explore the relative humidity (RH) conditions under which LLPS occurs and characterize the rate of evaporation of 3EG from well-mixed and LLPS particles as a function of RH. We show that the evaporation rates vary with RH due to changes in chemical activity, however no clear change in the dynamics following LLPS are observed. We interpret our observations using a thermodynamic model (AIOMFAC) coupled with an evaporation model and show that a significant increase in the activity coefficient of 3EG as the RH decreases, required for LLPS to occur, obscures a clear step-change in the evaporation rates following LLPS. By characterizing the evaporation rates, we estimate the composition of the organic-rich phase and compare our results to thermodynamic predictions. This study is the first to explore the connection between LLPS and the chemical evolution of aerosol particles *via* the evaporation of semi-volatile organic material. Ultimately, we reveal that the thermodynamics of non-ideal mixing are primarily responsible for the controlling both the rate of evaporation and the onset of LLPS, with LLPS itself having limited impact on the rate of evaporation in a fluid system. These results have significant implications for understanding and predicting the lifetime of aerosol particles, their effect on cloud formation, and the chemical evolution of multiphase systems by particle-gas partitioning and heterogeneous reactions.

## Abstract 2

Liquid–liquid phase separation (LLPS) is a thermodynamically driven process that occurs in mixtures of low miscibility material.

## Graphical abstract

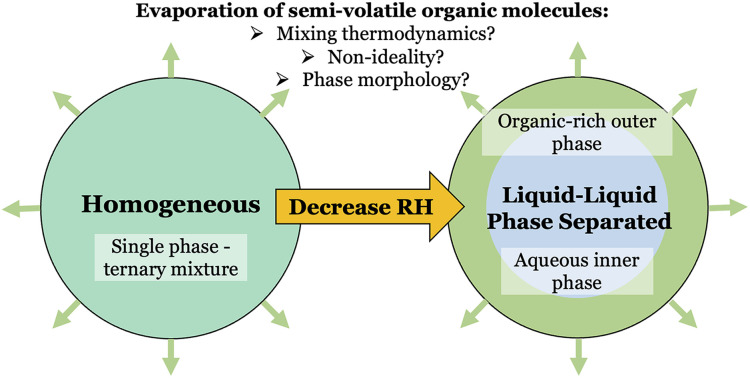

## Introduction

Liquid–liquid phase separation (LLPS) is an important chemical process that occurs when a mixture of low miscibility components separates to form two phases, thereby lowering the overall energy of the systems. As well as being important in biological systems, LLPS is a common occurrence in atmospheric aerosol particles. Atmospheric aerosols play important roles in the environment by interacting with solar radiation,^1^ providing nuclei for cloud and ice particle formation,^2^ interacting with gas phase oxidants,^3^ and impacting human health.^4^ Particles can contain a broad range of chemical compositions, including both inorganic and organic components, in addition to water in equilibrium with the gas phase relative humidity (RH).^5^ The interactions between organic and inorganic components in the particle can lead to unpredictable deviations in physical properties, such as changes in hygroscopicity and viscosity, that influence their effects in the atmosphere.^6–8^ These organic–inorganic interactions can also influence the phase state of the particle and, depending on the solubility and miscibility of the components present in the particle, can lead to LLPS.^9–11^ In aerosol particles, LLPS is in part controlled by the abundance of condensed phase water, determined by the RH, and the condition at which LLPS occurs on dehydration is known as the separation relative humidity (SRH). Phase-separation in particles can lead to changes in physiochemical properties that are not as readily predicable by extrapolation from homogeneous systems, including their heterogeneous reactivity,^12,13^ CCN activity,^7,14–16^ and optical properties.^17,18^ Thus, identification of LLPS and its incorporation into models for atmospherically relevant systems is of utmost importance.

Oxygen-to-carbon (O : C) ratios are used to give a general idea of the overall oxidation state of an organic molecule and can be used to predict whether it will lead to LLPS. While the forces governing phase separation are more complex than just overall oxidation state, organics with an O : C above 0.56 and below 0.80 are typically found to phase separate when mixed with ammonium sulfate (AS), a common atmospheric salt.^10,19,20^ Particles that have undergone phase separation can adopt core–shell morphologies, where an interior inorganic-rich core is coated by an outer shell of an organic rich phase, or partially engulfed morphologies, where the outer shell forms a lens that only partially covers the interior phase, allowing it to be exposed to the gas phase.^21–24^ A transition from core–shell to partially engulfed morphologies has been observed.^13,25^ To complicate things further, high and low polarity organics have also been found to separate into different organic phases in addition to an inorganic rich layer, resulting in three-phase aerosol particles.^26,27^ While atmospheric aerosol particles are typically more complex, lab-generated particles of AS with 3-methylglutaric acid (3MGA) and AS with 1,2,6-hexanetriol (HEX) have been observed to undergo LLPS in experiments performed on levitated particles, *via* Mie resonance spectroscopy, and droplets deposited on a substrate (usually a glass slide with a hydrophobic surface coating), *via* optical microscopy.^13,19^ In particles of AS and glutaric acid (GA), with an O : C of 0.8, LLPS is not clearly observed. However, as demonstrated by Shen *et al.*, even though AS-GA did not undergo LLPS, a notable increase in the reactive uptake of oxidants was observed, similar to phase separated AS-HEX, indicating that AS-GA particles were enriched in organic content near the surface, even if the particles did not exhibit full LLPS.^12^ High molecular weight polymer mixtures such as PEG400 and PEG1000 have also been observed to separate in AS-PEG particles.^11,28–30^ This is important as these PEG mixtures typically consist of polydisperse molecules whose chain lengths average 400 or 1000 g mol^−1^, in the cases of PEG400 and PEG1000 respectively, and indicates phase separation can still occur in organic–inorganic particles even if the organic molecules span a range of molar masses.

LLPS occurs through one of two mechanisms depending on the thermodynamics of the mixture and the mixing ratio of components. These two mechanisms can be identified in a phase diagram (see Fig. S1,† and *e.g.* Kucinski *et al.*^31^ and Ciobanu *et al.*^11^) and include a nucleation and growth mechanism, which requires overcoming an activation barrier, and a spinodal decomposition mechanism, which does not have an activation barrier.^10,32^ Due to the activation barrier associated with nucleation and growth, there is a metastable region in which LLPS is thermodynamically preferred but the particle can remain homogenous. This leads to hysteresis in the SRH and the RH at which the particle transitions back to a single phase on moistening (here referred to the as mixing RH (MRH)). The mechanism responsible for LLPS is challenging to measure experimentally and may vary with environmental factors such as the rate of change of RH and temperature. While MRH is much less frequently reported than SRH, these values are similar in SOA particles produced by alpha-pinene ozonolysis with and without ammonia,^29^ AS-PEG400,^11^ and AS-PEG400 mixed with C6-diacids.^30^ A hysteresis was found between SRH and MRH for AS-3MGA aerosols by lowering the pH, which resulted in a decreased SRH, while MRH remained consistently around 80%, possibly indicating a nucleation mechanism with a larger energy barrier or reflecting the influence of changing the composition rather than changing the mechanism of LLPS.^33^

The impact of LLPS morphologies on gas–particle partitioning is currently not well understood. Interactions in a core–shell particle between the gas phase and the particle will largely be governed by the composition of the outer phase after a particle undergoes LLPS, with the inorganic rich interior shielded from the gas phase.^10,28^ Thus, LLPS particles may exhibit different rates of evaporation of semi-volatile organics compared to well-mixed particles, and may experience increased reactivity towards gas phase reactive species, leading to the surface becoming more highly oxidized, especially if mixing in the particle is hindered.^21,34^ To the best of our knowledge, the impacts of LLPS on the evaporation of volatile organics has not yet bet explored, while only a handful of studies have explored changes in reactivity.^12,13^ The evaporation of volatile and semi-volatile organics from LLPS particles depends on the thermodynamics of the system, and measurements may offer broad insights into gas–particle partitioning and reactivity of LLPS particles.

In this work, we explore the onset of LLPS in levitated particles containing the semi-volatile organic compound triethylene glycol (3EG) and ammonium sulfate. 3EG was chosen as a proxy compound for semi-volatile organic molecules in atmospheric aerosols due to it having an elemental O/C ratio and chemical structure similar to those organic compounds in aerosols previously shown to exhibit LLPS, while being sufficiently volatile that evaporation rates would allow measurements over reasonable timescales. We characterize the separation RH and compare it to higher molecular weight PEG molecules. We go on to characterize the rate of evaporation of the 3EG as a function of RH spanning above and below the SRH. Incorporating thermodynamic modelling using AIOMFAC, we interpret the evaporation process in three ways through evaporation models that respectively assume a homogeneous ideal mixture, a homogeneous non-ideal mixture, and a LLPS model that assumes a core–shell morphology, with each phase behaving as if composed of a binary mixture. Overall, this simple system allows us to explore the applicability of some fundamental assumptions regarding the behavior of LLPS particles.

## Results and discussion

### Identifying LLPS in particles containing triethylene glycol with ammonium sulfate

**Fig. 1:**
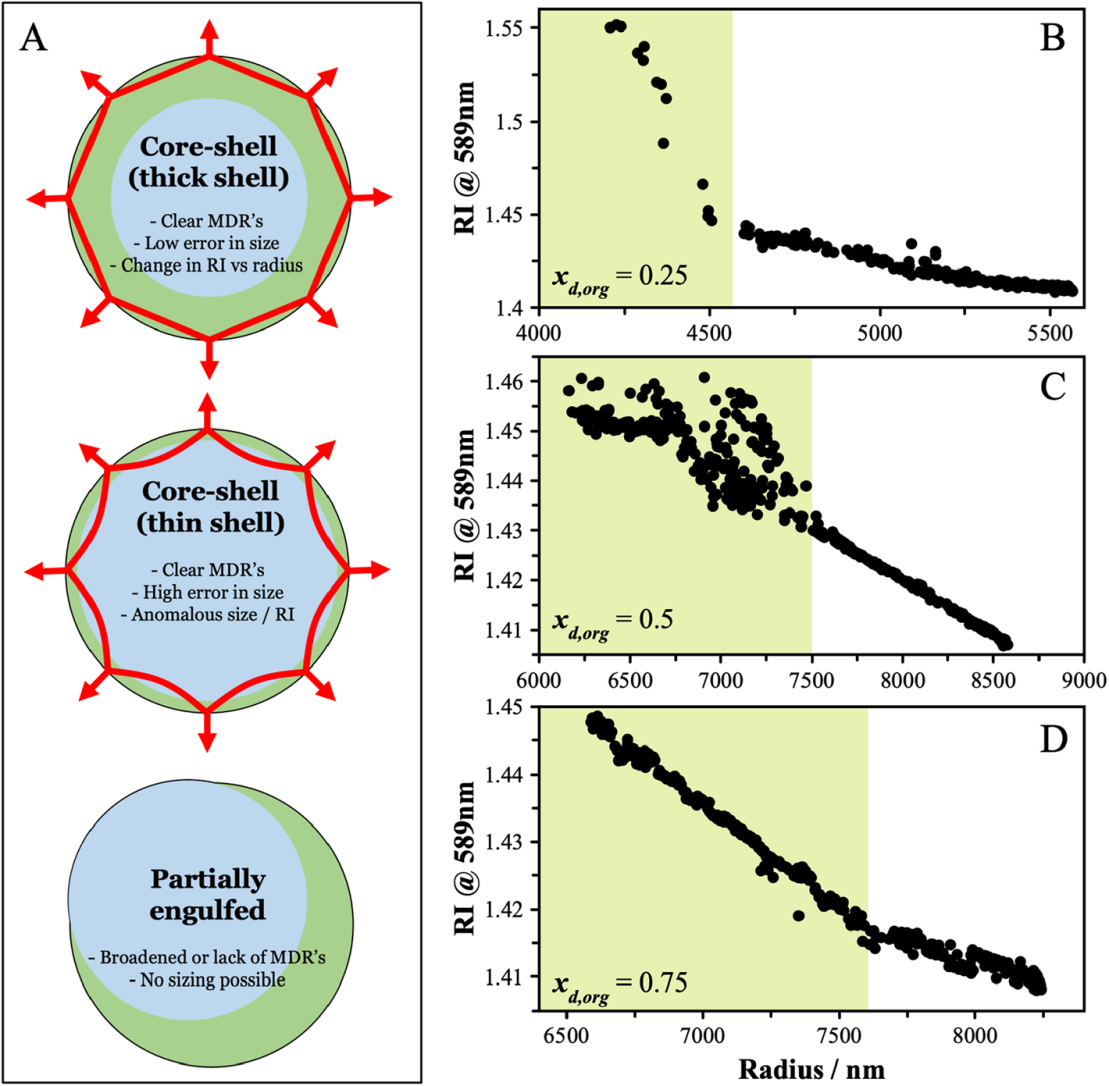
(A) Different morphologies will affect the MDR's and subsequent analysis, allowing LLPS to be identified. Green shading shows organic-rich regions and blue shading shown aqueous regions. (B) LLPS was identified in aqueous particles containing 3EG and AS from the discontinuity in the change in RI with radius as the RH was decreased, indicating core–shell morphologies with thick shells relative to the penetration depth of the MDR's (as shown in panel A – note that the position of the images in panel A do not correspond to the morphology observed in panels B–D). Three example starting particle compositions are shown, with dry organic mole fractions: (B) *x*_d,org_ = 0.25; (C) *x*_d,org_ = 0.5; and (D) *x*_d,org_ = 0.75. In each panel, the shaded region indicates where the particle was liquid–liquid phase separated. Note these measurements are obtained by decreasing the RH continuously from a starting level of ∼80% to 40% over a time span of around 2000 s.

Triethylene glycol (3EG) has an O/C ratio of 0.66 and is similar to other compounds that have been identified to undergo LLPS. To identify the onset RH of LLPS in aqueous 3EG/AS particles, samples were levitated in a linear quadrupole electrodynamic balance (LQ-EDB) at high RH and exposed to a gradually decreasing RH over around 500 s, a timescale chosen to limit the extent of evaporation of the semi-volatile 3EG. The methods adopted here are described in detail in the Methods section and the ESI.† Briefly, we measure the wavelength positions of the morphology-dependent resonances (MDR's) in the particle using Mie resonance spectroscopy, which are a function of the size and refractive index (RI) of the particle. For homogeneous spherical particles, the MDR's allow the size and RI to be determined with a high accuracy and precision.^35^ In LLPS particles, the existence of a liquid–liquid boundary may interfere with the MDR's and lead to characteristic changes in the resulting spectra. Depending on the morphology (core–shell *vs.* partially engulfed) and the thickness of the organic shell, the MDR's will be affected in a few different ways, as depicted in Fig. 1A. In general, from the manner in which the measured RI and radius vary as the RH is decreased, we can identify the onset of LLPS. Some examples of the evidence that points towards LLPS are shown in Fig. 1B–D, showing the characteristic breakdown in the variation of RI with radius, attributed to the formation of a LLPS particle with an organic-rich shell. The continued ability to obtain the size and RI from the Mie resonance spectra with a low error indicates one of two scenarios. One scenario is that the particle adopts a spherically symmetric core–shell structure in which the MDR's are entrained solely within the organic-rich phase, due to their shallow penetration depth (thick shell configuration). Alternatively, if the RI were very similar between the aqueous and organic-rich phases, the boundary would have limited impact on the observed spectra, thus even if the MDR's crossed the boundary (thin shell configuration), there would be little impact on the spectra and interpretation. For the purposes of these measurements, either scenario results in both clear identification of LLPS and reliable sizing data following LLPS. For partially engulfed morphologies and emulsions, the spectra breakdown as the MDR's cannot be supported by asymmetrical particle shapes leading to a breakdown in the sizing process. The features that allow LLPS to be identified are described in more detail in the ESI.†

**Fig. 2:**
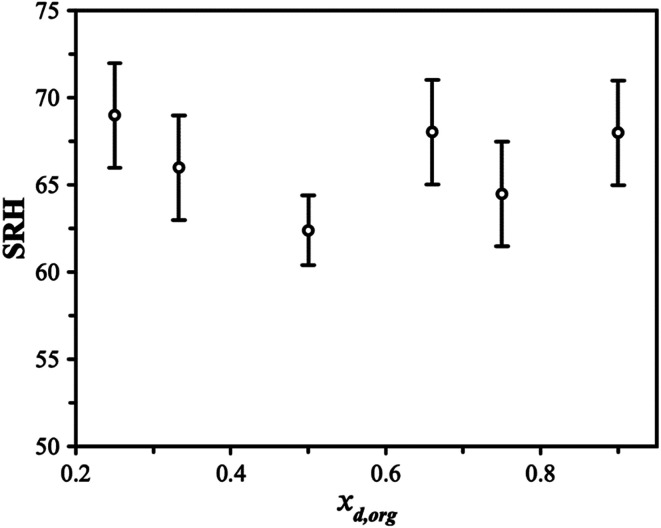
The separation RH (SRH) upon dehumidification at 293 K is shown as a function of the starting dry organic mole fraction (*x*_d,org_) in the particle.

The SRH's observed for this system do not show a clear dependence on the organic dry mole fraction, *x*_d,org_, as see in Fig. 2. While characterization of a precise phase diagram of this system is not the main focus of this work, the lack of a strong dependence on measured compositions is broadly consistent with previous work on higher molecular-weight PEG molecules (PEG400).^36^ The uncertainty in the SRH at a given dry composition may arise due to the different mechanisms for LLPS that may be in play, as discussed briefly in the introduction. LLPS occurring *via* a spinodal decomposition at compositions close to the critical point will not show significant variability between measurements and will be purely under thermodynamic control. For a nucleation and growth mechanism, a metastable regime is possible and LLPS occurs with an energy barrier. The extent of the variability in SRH will depend on the size of the energy barrier and other factors, such as the rate of RH change and the size of the particle. For example, in the recent work of Huang *et al.*, both size and timescales were found to lead to variability in the SRH.^37^ Measurements of remixing were not performed due to the evaporation of the 3EG leading to the particles changing in composition significantly on downward and upward RH trajectories (affecting *x*_d,org_). The thermodynamic factors regulating LLPS in this system are explored in more detail later as we compare the observations with predictions using thermodynamic modelling.

### Evaporation of triethylene glycol from mixed particles

**Fig. 3:**
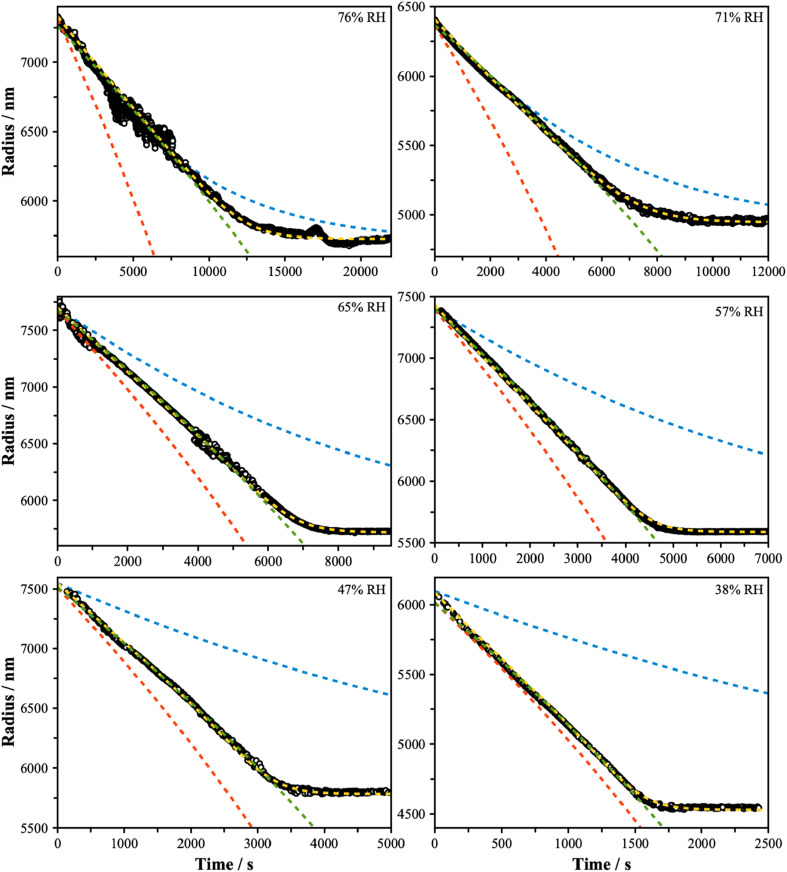
Evaporation measurements were performed over a range of RH conditions, as indicated, and span above and below the separation RH (∼65%). In each panel, the blue dashed line shows the predicted trend assuming ideality (model 1 – mole fraction scaled evaporation rates), the yellow dashed line assumes a well-mixed phase that exhibits non-ideality (model 2), with fitted activity coefficients reported in Fig. S4,† and the red dashed line assumes a core–shell particle with a binary organic-rich shell (model 3). The green dashed line is a fit to 
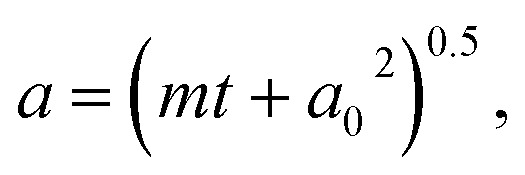
 with radius *a*, starting radius *a*_0_ and slope parameter *m*, equivalent to a linear fit of radius-squared *versus* time data for the early part of radius change measurements.

Subsequent measurements were performed to characterize the rate of evaporation of 3EG from particles containing a mixture of 3EG and ammonium sulfate. Particles with a dry equimolar starting composition with respect to undissociated AS (*i.e. x*_d,org_ = 0.5, *w*_d,org_ = 0.53) were levitated in the LQ-EDB and the Mie resonance spectra were recorded as the particle decreased in size due to evaporation. Measurements were performed in triplicate spanning the whole evaporation process. Individual measurements were made in RH conditions that varied in the range from 86% to 38% to span the conditions before and after the onset of LLPS. Fig. 3 shows representative radius *versus* time data for each RH condition explored in this work. In each case, the particles reach a steady size, attributed to the complete evaporative loss of 3EG and producing a particle consisting of AS and water only. As the RH is decreased, the rate of evaporation is increased, consistent with the increase in effective vapor pressure of the organic component expected as the water mole fraction in the particle is reduced. Furthermore, the evaporation rate, often quantified by the change in radius-squared with time, decreases throughout the measurement, consistent with the continuous decrease in the mole fraction of organic in the particle. Interestingly, for measurements at low RH, the size trend exhibits a dependence that resembles the evaporation of a single component system – that is, the radius-squared evaporation rate varies in a linear manner with time. Given that these are not single component particles, this observation points to some interesting thermodynamics controlling the rate of evaporation that may be connected with LLPS, as explored further using three evaporation models described in the subsequent sections.

The factors that control the rate of evaporation in a mixed system that exhibits hygroscopic growth are the vapor pressure of the semi-volatile component in its pure state 
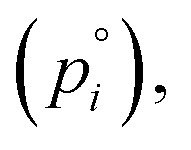
 which is only a function of temperature, the activity of the component in the mixture, and the amount of water associated with each evaporating molecule. Our previous work, as well as several other studies, have reported on the vapor pressure of pure 3EG. At 298 K, Krieger *et al.*^38^ report the vapor pressure to be 
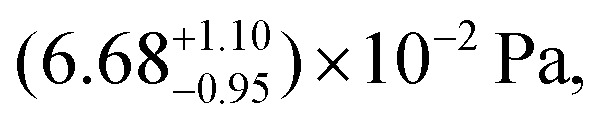
 with an enthalpy of vaporization of 84.3 ± 1.9 kJ mol^−1^. Our earlier measurements at a slightly lower temperature gave a vapor pressure of (5.1 ± 0.1) × 10^−2^ Pa, consistent with a lab temperature of ∼295 K. In this work, evaporation measurements on pure 3EG (see ESI†) across a range of RH indicate a vapor pressure of (3.0 ± 0.1) × 10^−2^ Pa at a lab temperature of 292 K. We use our newly measured value in this work to ensure consistency with respect to the environmental conditions. The activity of the organic molecules in the mixture is more difficult to quantify and we adopt the thermodynamic model AIOMFAC to predict the composition of the particles, as described in the Methods.^39,40^ To simplify the computation in the evaporation model, we use the ZSR assumption with the AIOMFAC outputs for individual binary systems containing AS with water and 3EG with water to predict the properties of the ternary mixture. While this approach discounts the molecular interactions that might lead to deviations from the ZSR model, it simplifies the application of mixing thermodynamics in the evaporation models where the composition is constantly changing. We compare the results of the ZSR approach and the full application of AIOMFAC for the predicted composition of an equimolar mixture in Fig. S3† and show that the differences are relatively small and their influence on the interpretation of the results should be negligible. The mole fractions determined using this approach may be used in conjunction with activity coefficients, either fit to the data or predicted by a model, to account for non-ideal interactions among all solute and solvent species, and also allow for the amount of co-evaporating water associated with the evaporating semi-volatile molecules to be incorporated in the model.

The evaporation model we adopt here is based on an isothermal continuum mass flux model in which the limiting step in the mass transport process is gas phase diffusion. In this framework, the total mass flux from a particle with radius *a* is given by:1
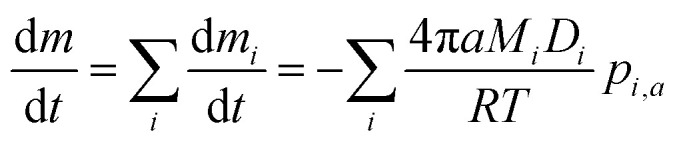
where *m*_*i*_ is the mass of species *i* with molar mass *M*_*i*_ and density *ρ*_*i*_, and *p*_*i*,*a*_ is the liquid-state (equilibrium) vapor pressure of *i* over the mixture, determined from 
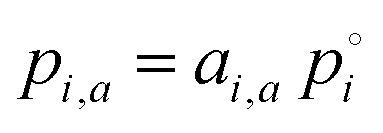
 (modified Raoult's law) with the activity *a*_*i*,*a*_ set equal to the product of the mole fraction *x*_*i*,*a*_ and activity coefficient *γ*_*i*,*a*_, and liquid-state pure component vapor pressure 
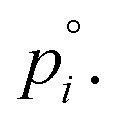
 Note that the subscript “*a*” indicates these parameters are associated with the particle (of radius *a*). When the evaporation of component *i* at constant RH leads to an additional loss of some amount of water associated with the hygroscopicity of that component, this will lead to additional mass loss from the particle. To account for this, we need to determine the amount of water associated with an evaporating unit mass of each component at a given RH level, such that the total mass flux is:2
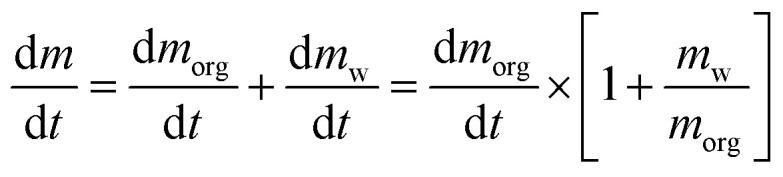


In this formulation, we have replaced the mass flux of water with the product of the mass flux of organic and the mass ratio of water to organic. The mass ratio may be determined by invoking the ZSR relation, which states that the amount of water associated with a solute in a binary solution, at a given RH, will be the same relative amount of water associated with that solute in a ternary solution. If the mole fraction of component *i* in a binary mixture with water is given by *x*_*i*_, we can replace the mass ratio term from the square bracket with a function of the molar mass and mole fraction, and combine eqn (2) and (3) to yield the full mass flux equation:3
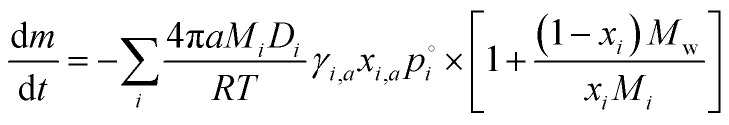


**Fig. 4:**
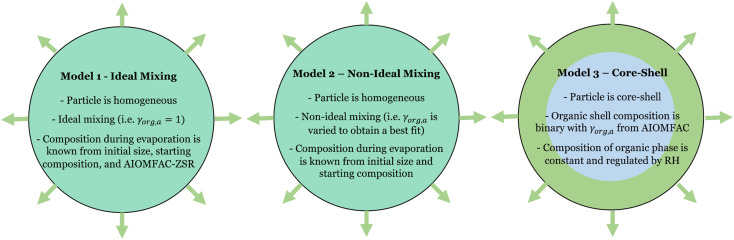
Schematic description of the model configurations adopted in this work.

Here, we have included the activity coefficient and the mole fraction of *i* in the ternary particle (*γ*_*i*,*a*_ and *x*_*i*,*a*_) which include the dissociation of the inorganic species as well as the composition in the particle. These are distinct from the binary mole fraction (*x*_*i*_) which refers only to the composition of the mixture of *i* and water at the same RH. We can apply this evaporation model using different assumptions, as discussed in subsequent sections, and summarized in Fig. 4.

#### Model 1: ideal mixing

When we apply the evaporation model built using eqn (3), using our measured 
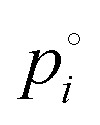
 for 3EG and the gas phase diffusion parameters reported in Krieger *et al.*,^38,41,42^ and assume that the activity coefficients of the organic component in the ternary mixture are unity, then we obtain the predictions shown by the blue dash lines in Fig. 3. At high RH, this leads to relatively close agreement between the measurement and model, while at low RH the model is a significant underestimate of the evaporation rate. Further, the shapes of the predicted curves differ substantially, with much clearer curvature exhibited by the predictions than by the experimental data. These comparisons point towards non-ideal mixing in the system that leads to evaporation rates that are influenced by activity coefficients that deviate substantially from unity.

#### Model 2: non-ideal mixing

Here, we force the system to exist as a single liquid phase (*i.e.*, a homogeneous particle). Within this model framework, both the composition of the particle and the pure component vapor pressure are known. Thus, only the activity coefficient of the evaporating component can vary, allowing us to evaluate this parameter across the measurements. Based on the AIOMFAC predictions of the activity coefficient of 3EG as a function of its mole fraction in a ternary solution (Fig. S4†), a reasonable functional fit was selected based on an offset exponential decay with three empirical parameters (*γ*_org_ = *Ae*^−*kx_org,a_*^ + B). The empirical fit parameters (*A*, *k*, *B*) were varied to minimize the mean-squared difference between the measurements and predicted evaporation trend.

The results of the evaporation modelling are shown in Fig. 3 (yellow lines) and are capable of fully describing the evaporation of the organic in all cases at all RH. The activity coefficients of 3EG are shown in Fig. S4† as a function of the mole fraction of 3EG in the particle. These all show a steep increase as the organic fraction decreases, as well as increasing with decreasing RH. These trends are consistent with the predictions from the AIOMFAC model for the ternary mixture when forcing a single phase, although the magnitudes differ which may be attributed to the parameterization of the relevant functional group parameters in the model, as described later. It should be noted that a direct comparison between the experiment and AIOMFAC predictions here are limited as the system is known to not exist as a single phase, and indeed the AIOMFAC activity coefficients increase substantially after the point at which LLPS is expected. However, these data do clearly indicate that a continuously varying set of activity coefficients is fully capable of describing the dynamics of the system before and after LLPS, indicating that it is the thermodynamics of the mixture that regulates both the evaporation behavior and the onset and/or occurrence of LLPS.

Owing to the independent evidence of LLPS in this system, we can explore the evaporation trend that might be expected for a two-phase particle in which the outer phase is an organic-rich shell. The Mie resonance spectra show clear MDR's consistent with a spherical particle, indicating that the LLPS particles adopt a core–shell morphology. This can significantly simplify the analysis of the evaporation rates as the particle remains spherically symmetric.

#### Model 3: core–shell LLPS

If we assume we are dealing with a core–shell particle in which the outer shell consists only of 3EG and its associated equilibrium water content as a function of RH, then we can simplify the mass flux equation and treat the system as if it were a binary mixed particle with an inert core. A binary mixed particle undergoing isothermal evaporation in the continuum regime exhibits a linear radius-squared dependence on time, which is exactly what is observed in these measurements for most of the evaporation trajectory, until the plateau region is reached where the size levels off and the particle has lost all of the organic material. The integrated form of eqn (1) provides an analytical solution that decouples the evaporation rate from the size of the particle:4
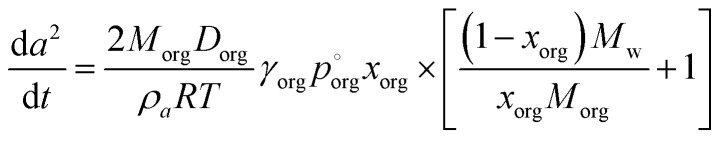
where we have replaced subscript *i* with org and treat the system with a binary aqueous organic composition. As before, the term in the square bracket accounts for the concomitant loss of water when an organic molecule evaporates. In this case, *x*_*i*,*a*_ = *x*_*i*_ = *x*_org_. Taking the activity coefficient of the organic component from the AIOMFAC model for the binary system, we can predict the evaporation rate under these assumptions, shown by the red dashed line of Fig. 3.

**Fig. 5:**
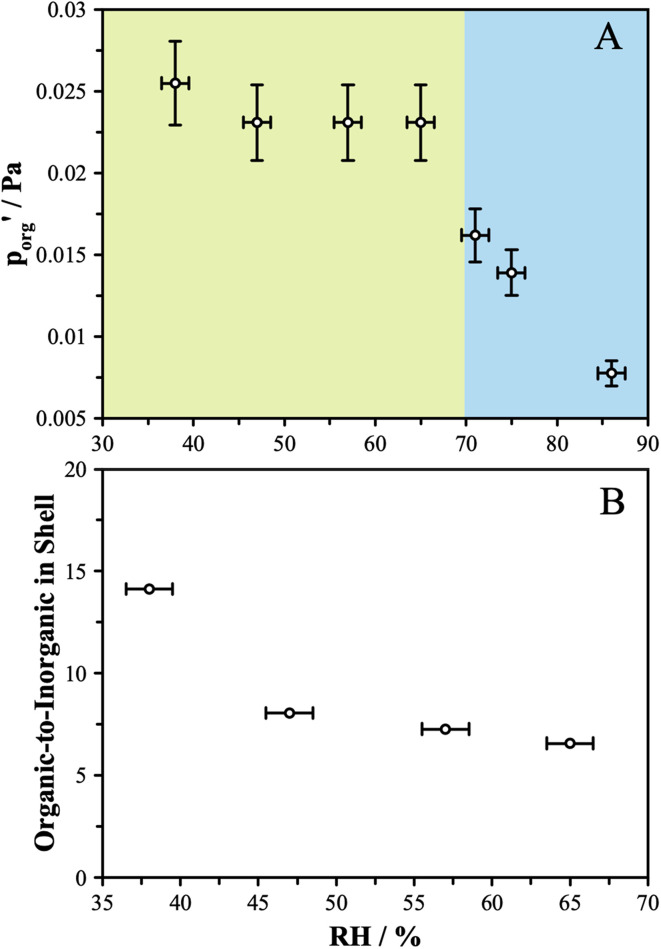
(A) Under the assumptions of core–shell behavior and an organic-rich phase that is modelled with a binary composition, the effective vapor pressure was determined from the slope of radius-squared *versus* time for the linear portion of the evaporation trajectories. The blue shaded region shows where the particle is assumed to be well-mixed (and thus this assumption fails) and the green shaded region shows where the particle is assumed to be phase-separated with a core–shell morphology. (B) In order to achieve closure between the pure-component vapor pressure and the expected vapor pressure, the organic-rich phase must include some AS and additional water which collectively lower the activity of the organic component. Assuming the activity coefficient of the organic remains the same as in a binary mixture, the organic-to-inorganic mole ratio in the shell was calculated. As described in the text, this assumption of activity coefficients likely introduces error as AIOMFAC modelling indicates that the presence of AS increases the activity coefficient of the organic.

This model significantly over-predicts the evaporation rate at high RH, where the particles are homogeneous, as it omits any contribution from the AS and the water associated with the AS, which dilute the particle and thus lower the actual mole fraction as well as the activity of 3EG in the homogenous case. In the RH range where LLPS is expected, this model only slightly overpredicts the evaporation rate. Instead of fixing the value of 
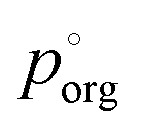
 to 3.0 × 10^−2^ Pa as in the other models, we can use the measured evaporation rate to solve for 
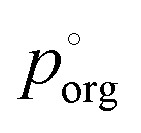
 under the assumption that the particle behaves as a binary mixture. We refer to this value as 
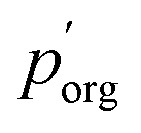
 to delineate it from the actual pure-component vapor pressure, and show it in Fig. 5A as a function of RH. We can identify two regions of interest based on whether the particle is homogeneous or LLPS. For the homogeneous particle, 
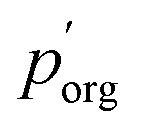
 will be a large underestimate compared to the pure-component value of 3EG due to the presence of the AS and additional water that is not accounted for by the other assumptions in this model. As the RH decreases, 
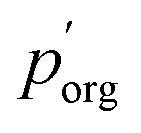
 becomes less of an underestimate, and in the region where we detected core–shell particles, it becomes approximately constant as a function of RH.

We can use these data to estimate how much AS might be present in the organic-rich phase to give the measured evaporation rates, as we know what the pure component vapor pressure must be in this case. Using 3.0 × 10^−2^ Pa as the value of 
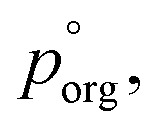
 and the AIOMFAC predictions for the amount of water associated with AS in a binary system at the same RH, we can estimate the mole ratio of organic molecules to undissociated inorganic molecules in the shell of the particle by applying the ZSR relation, shown in Fig. 5B. We report these data only for the measurements in the LLPS region, as the assumptions that go into the calculation break down if LLPS is not present. Based on the AIOMFAC predictions, the presence of small amounts of AS in the organic-rich phase will likely increase the activity coefficient of the organic while reducing its mole fraction. Thus, the inferred compositions reported in Fig. 5B should be considered representative within the assumption underlying the ZSR relation with respect to the addition of AS to the organic phase (*i.e.* the activity coefficient of the organic remains unchanged with respect to the binary case). Table S2† provides a breakdown of the calculations contributing to these estimates.

### Thermodynamic interpretation of experimental observations *via* AIOMFAC modelling

Up to this point we have relied upon AIOMFAC calculations and ZSR to predict the composition of the particles assuming a single phase is present, or in the case of LLPS, that a binary mixture is present in each of the two phases. However, the AIOMFAC-LLPS model is capable of predicting the onset of LLPS in mixed organic–inorganic systems and has been successfully applied in several cases. Of note, for mixtures including other (poly)ethylene glycols, such as PEG400 and higher molecular weights, AIOMFAC predictions of LLPS with AS have been shown to perform well compared to experiments.^11^ The oxyethylene functional group parameters used in AIOMFAC as part of this work was previously introduced in work exploring the hygroscopic growth of high molecular weight PEG's. It has been shown to be effective in predicting the hygroscopic growth factors measured experimentally for PEG200, PEG1000, and PEG10000.^28^ However, it should be noted that while 3EG is somewhat equivalent to a hypothetical PEG150 and should exhibit similar behavior, the average number of oxyethylene groups per molecule is two for 3EG, *versus* three for PEG200 and around 22 for PEG1000. Thus, the smaller molecule will be more polar and its behavior will be weighted more towards the interactions of the OH groups with water and AS rather than with the oxyethylene groups.

The predicted phase diagram for a ternary mixture of 3EG, AS and water is shown in Fig. S5.† There are several interesting regions of this phase diagram, described in more detail in the ESI,† that may be compared with our results. Firstly, the predicted onset of LLPS is around 85% RH for the equimolar mixture, compared to separation occurring in the range from 60 to 70% observed experimentally. Based on this phase diagram, the composition of each phase can be predicted. Initially, at the SRH for an equimolar mixture, a particle with a starting organic-to-AS ratio of 1 will separate with an organic-rich phase that has an organic-to-AS mole ratio of ∼1.3, increasing rapidly as the RH decreases to yield almost pure organic in the organic rich phase at below 80% RH. These observations are consistent with our predictions that some inorganic remains in the organic-rich phase, although quantitative differences arise, in part due to the assumptions that the activity of the organic in the organic-rich phase may be given by the activity predicted by the binary organic and water system. However, the activity coefficients of the 3EG in the organic-rich phase predicted by this model are consistent with the binary activity coefficients used in model 3, indicating the mixing thermodynamics in the organic-rich phase are minimally affected by the presence of small amounts of AS. Finally, the phase diagram indicates the phase transition range to be relatively flat over the composition range, which is also supported by our measurements. The phase diagram suggests that as the organic material from the particle evaporates, the onset of LLPS does not change to a measurable degree, and for particles that are already LLPS, the composition of each phase will remain broadly the same. This is consistent with the observed radius-squared dependence of the evaporation rate for the LLPS particles, as the evaporating phase retains a nearly constant activity of the organic.

The differences between the AIOMFAC-LLPS predictions may be attributed to the interactions between the ions and oxyethylene groups and/or OH groups. If 3EG is relatively more miscible with AS than its higher molecular weight counterparts (PEG400 *etc.*), then a lower SRH would be expected, and the organic-rich phase could support more AS, as inferred from our measurements. Increased miscibility would also change the phase diagram, lowering the LLPS boundary and narrowing the width of the LLPS region, allowing for more AS to exist in the organic rich phase. While we do not have sufficient data to produce a fully accurate phase diagram, our results indicate that the SRH is approximately constant across the compositional range explored, albeit with high variability as captured by the error bars shown in Fig. 2. This behavior is consistent with the middle of the composition range in PEG 400/AS mixtures and is predicted by the AIOMFAC model.^11^

## Conclusions

Through measurements on the onset of LLPS and the evaporation behavior of particles containing AS and triethylene glycol, we begin to unravel the thermodynamic implications of LLPS. We identify LLPS at a lower RH than previous work on higher molecular weight PEG molecules, which is attributed to the increased miscibility of 3EG due to the great proportional of OH groups per molecule. The particles form core–shell morphologies, as evidenced by the retention of clear peaks in the Mie resonance spectra. Notably, from measurements on the evaporation of 3EG from these particles, we do not see a clear step-change in the evaporation rates going from well-mixed to core–shell morphologies. This is rationalized from the activity of the organic in the homogeneous mixture and the LLPS particle. For LLPS to occur, the activity coefficient is necessarily high, indicating the organic is not energetically stable in solution relative to an ideal mixture. Thus, as the RH is lowered and the SRH is approached, the activity of the organic is already increasing significantly, giving rise to faster rates of evaporation than would be predicted assuming ideality. At the point of LLPS, while the composition shifts significantly and the outer shell becomes much more enriched in organic molecules, the activity of the organic remains approximately the same and its activity coefficient decreases.

Ultimately, these measurements demonstrate some of the limitations of the assumption that LLPS will lead to changes in the chemical properties of the particle. While it is clearly true that geometrically the outer layer is more exposed to the gas phase, it is the chemical activity of the organic molecules in the particle that determine how they interact with the gas phase, evident in this work with respect to liquid–vapor partitioning. For chemical reactivity, similar arguments could be made for why reaction rates are not drastically increased following LLPS – it is the activity of the reactant that is important, rather than its concentration. If gas phase reactants, such as radical species are able to adsorb and sample the surface region of the particle, they will be similarly able to find an organic molecule in an LLPS particle as in a homogeneous particle, assuming adsorption times are sufficiently long for diffusion to bring reactants together. Going further, in particles that will experience LLPS, the low miscibility will likely lead to organic molecules preferentially residing at the interface even in homogeneous particles, further negating the extent of any step-change due to the occurrence of LLPS. This same argument may be made with respect to the influence of LLPS on cloud formation.^16^ That is, particles that exhibit LLPS will contain organic compounds that exhibit low miscibility and high surface activity, leading to depression of the surface tension and changes in the cloud formation activity of the particles.

Although more work must be done on both the thermodynamics of LLPS systems and their resulting chemical behavior, this work demonstrates that in systems that exhibit LLPS, it is non-ideality that is the determining factor in the chemical evolution. Broadening this conclusion further, we argue that observations of chemical effects in LLPS particles may be incorrectly ascribed to phase separation and are instead directly caused by the same underlying thermodynamics that also lead to phase separation. That is to say that LLPS, gas–particle partitioning, bulk–surface partitioning, hygroscopicity, CCN activity, evaporation rates, *etc.* are all controlled by the underlying chemical thermodynamics. However, it is important to consider cases in which LLPS will have a direct impact on chemical evolution, such as through diffusion limitations due to the formation of organic-rich phase with a much higher viscosity than the homogeneous mixture. For example, in Zhou *et al.*, the formation of a viscous organic crust following LLPS hinders the diffusion of ozone leading to slow heterogeneous reaction kinetics.^43^ Similar effects may be observed that slow evaporation rates and a consideration of the viscosity of each phase and the particle morphology may be necessary. Further work is required to explore the connections between these processes and the influence of highly viscous states that might form after LLPS in some systems.

## Methods

### Sample preparation

Chemicals were acquired from Sigma Aldrich (>99% purity) and solutions were prepared without further purification. Ternary solutions of AS and 3EG were made at organic-to-inorganic mole ratios of 1 : 1, 1 : 2, 1 : 3, 2 : 1, and 3 : 1 (*x*_d,org_ = 0.5, 0.33, 0.25, 0.67, 0.75, respectively) at a total mass concentration of 10 g kg^−1^ of water. Individual droplets of around 25 μm in radius were generated from a Microfab MJ-ABP-01 microdroplet dispenser in the presence of a positively charged induction voltage plate. The induced net negative charge on the droplets allowed them to be confined in a linear quadrupole electrodynamic balance (LQ-EDB).

### Particle levitation and environmental control

The LQ-EDB has been described in detail in our previous work.^41,44^ Briefly, single droplets introduced into the LQ-EDB are confined to the center of the linear quadrupole axis by an AC electric field and balanced vertically against gravity and a downward flowing gas (at 200 sccm) by a negatively charged DC plate. Dilute aqueous droplets produced from the dispenser rapidly lose water to equilibrate to the RH within the chamber, yielding particles that were around 7 μm in radius when trapped at 80%. Dry and humidified N_2_ gas flows were mixed by a mass flow controller to control the RH in the chamber and calibrated capacitance RH probes were used to monitor the RH inside the chamber. Experiments were performed either at a fixed RH (evaporation measurements) or with a gradually decreasing RH (LLPS identification).

### Mie resonance spectroscopy

Mie resonance spectroscopy (MRS) was used to characterize the size and refractive index (RI) of levitated particles in the manner described in our previous work.^41^ Briefly, a red LED was used to illuminate the levitated particle and an ocean insight HR4000 spectrometer was used to sample the backscattered light. Spectra were collected every 1 s and the morphology dependent resonances (MDR's), identified as sharp peaks in the spectra, were used to determine the size and RI of the levitated particle using the MRFIT algorithm developed by Preston and Reid.^35^ Experimental spectra were subsequently compared to simulated spectra to confirm proper sizing by comparing the spectra shape and width between experiment and theory. A green laser (532 nm) was used to illuminate the droplet to verify one was in the trap and to balance it if the droplet could not be balanced by the PID feedback look from red LED illumination alone. The green laser did not serve a spectroscopic purpose in this study. MRS also provides a means of identifying the onset of LLPS in levitated particles, as detailed in the ESI† and in previous work.^13^

### AIOMFAC thermodynamic modelling

We applied the AIOMFAC model to understand the thermodynamics of mixed solutions and predict their behavior and phase.^39,40^ Briefly, this model allows the activity and activity coefficients of all components in a mixture to be calculated, using a group contribution approach for organic molecules. Recently, this model has been further developed to probe and predict the onset of LLPS, with the ability to generate full phase diagrams across composition and environmental conditions.^32,45^ The AIOMFAC model was used here to predict the composition and activity coefficients of binary mixtures, ternary mixtures, and the full phase diagram including LLPS.

## Data availability

Relevant data is available in the ESI† and on request.

## Author contributions

J. F. D. conceptualized the project, J. M. C, B. S., and J. S. performed the measurements, J. F. D. and J. C. analyzed and interpreted the data, along with contributions from A. Z., and J. F. D, J. C., and A. Z. wrote the manuscript.

## Conflicts of interest

The authors declare no conflicts.

## Supplementary Material

SC-015-D3SC05164A-s001
