# Supplementary material for: Probing the evaporation dynamics of semi-volatile organic compounds to reveal the thermodynamics of liquid–liquid phase separated aerosol
Source: Chem Sci. 2024 Jan 17;15(8):2963–74. doi: 10.1039/d3sc05164a (PMC10882461; doi:10.1039/d3sc05164a)
Supplement: SC-015-D3SC05164A-s001 [file SC-015-D3SC05164A-s001.pdf]

Electronic Supplementary Material (ESI) for Chemical Science.
This journal is © The Royal Society of Chemistry 2024

Supplementary Information

Probing the Evaporation Dynamics of Semi-Volatile Organic Compounds to Reveal the Thermodynamics of Liquid-Liquid Phase Separated Aerosol

Jack M. Choczynski[1], Bilal Shokoor[1], Jorge Salazar[1], Andreas Zuend[2], James F. Davies[1]*

1. Department of Chemistry, University of California Riverside, Riverside CA, USA
2. Department of Atmospheric and Oceanic Sciences, McGill University, Montreal, Quebec, Canada

* *Correspondence to:* James F. Davies (jfdavies@ucr.edu)

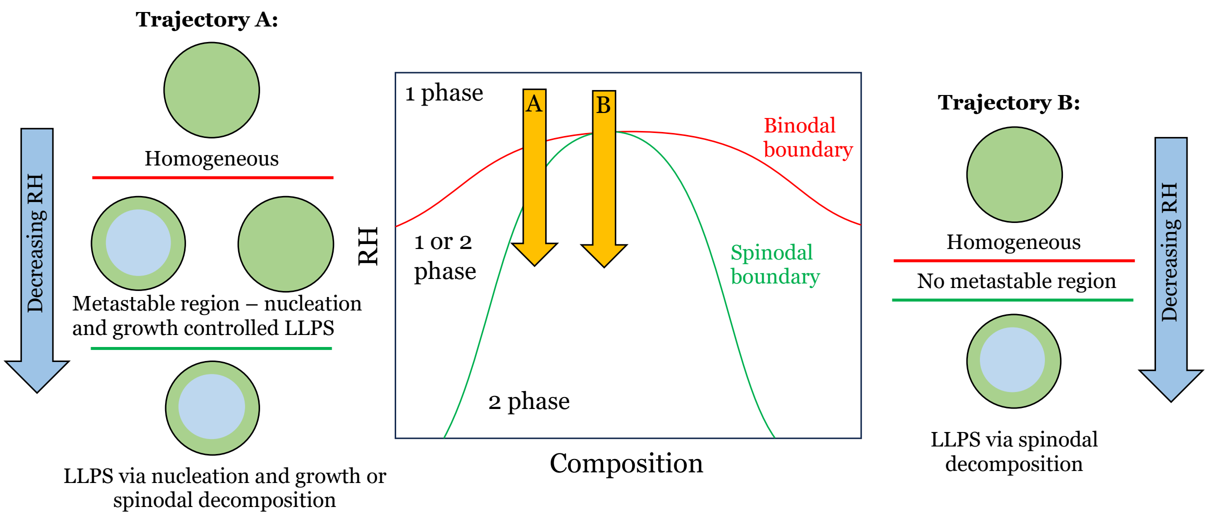

Figure S1: Schematic phase diagram indicating the two kinds of LLPS trajectory.

1. Identifying LLPS using MRS

To demonstrate the identification of LLPS using MRS, we performed measurements on ternary particles containing components that are known to experience LLPS. Solutions of 3-methylglutaric acid with AS and 1,2,6-hexanetriol with AS were prepared in the same manner as described for 3-EG in the main text. These components were selected as a nonvolatile organic and serve to validate spectroscopic identification of LLPS. Particles were dehumidified from 85 down to 40% RH in small steps to identify their SRH. 3-Methylglutaric acid was found to separate at $74 \pm 2\ \%$ RH, while 1,2,6-hexanetriol was found to separate at $72 \pm 1\ \%$ RH. These were determined by analyzing the dependence of the RI on the radius, as described below. The SRH ranges we report here are slightly lower than those reported by microscopy on a substrate, but that is likely due to the substrate surface inducing nucleation at an RH slightly above where a suspended droplet would separate that was not in contact with a surface.

LLPS is apparent from MRS data in one of two ways. The first is from a breakdown in the shape of the WGM peaks in the spectra. This occurs when either an additional interface scatters light, or if the particle deviates from a sphere. The extent of the breakdown in the spectrum usually indicates core-shell or partially engulfed morphologies. In the case of core shell morphologies, the spherical shape a retained and LLPS can be identified from a breakdown in the sizing algorithm or anomalous changes in how the radius and refractive index vary. To demonstrate these factors, samples were exposed to a steadily decrease RH starting at 85% and ranging down to 40% RH.

Figure S2A shows the radius vs refractive index for particles containing 3-methylglutaric acid and AS in a equimolar mixture as the RH is decreased at 0.5% per 30 s. The onset of LLPS here is clear indicated by the change in the slope of the plot and corresponds to the change in the composition of the phase primarily probed by the WGM's. When the RH is increased, a similar plot is observed showing remixing. Once LLPS is reached, there is also an increase in the error in the sizing results, and the variability in the size and RI increases. Typically, for core-shell particles, WGMs are still present, and the spectra are sizable under assumptions of a homogeneous structure. When lowering the RH further below the SRH, a near complete breakdown of the WGMs in the spectra is observed (Figure S2B).While some sharp peaks

remain, the spectra are too noisy to size here and may indicate the particle has now become a partially engulfed structure as the sphericity of the core-shell has been disrupted.

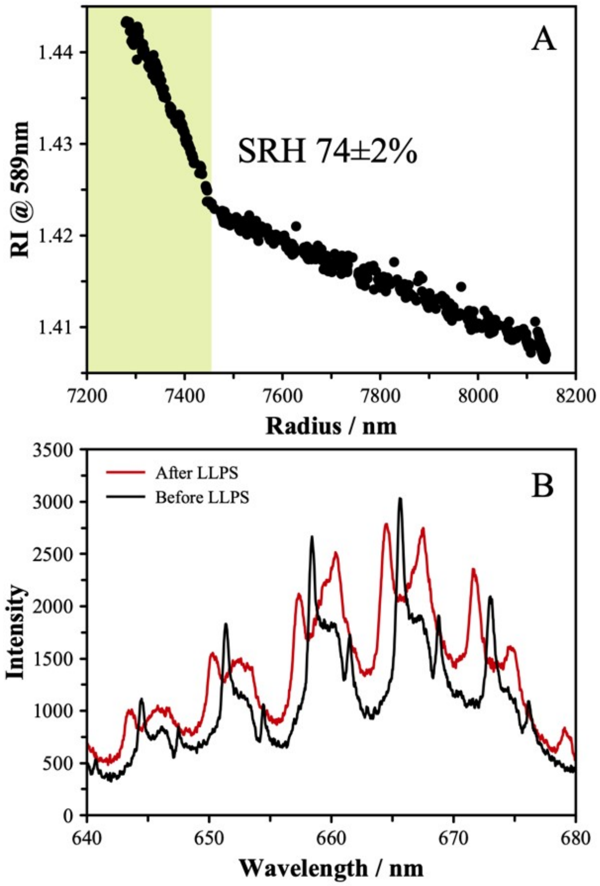

Figure S2: (A) The RI is shown as a function of the particle size as the RH was decreased from around 80% to 40%. The green shaded region indicates the range over which the particle exhibits LLPS, identified by the change in the slope of RI with radius. (B) The Mie resonance spectra are compared for a particle before LLPS and after. The breakdown in the sharpness of the peaks indicates the particle is losing spherical symmetry.

2. RH-Dependence of the Evaporation of Pure 3EG Particles

Measurements on the evaporation of particles containing pure 3EG as a solute were also performed to validate the binary thermodynamic data and establish the most accurate value for the saturation vapor pressure. The measurements are presented in Table S1.

Table S1: The pure component vapor pressure was determined from measurements on the evaporation of 3EG as a function of RH. For each RH, the AIOMFAC model was used to determine the composition and activity coefficient of the organic. The measured evaporation rate was used to determine p_{eff} and the composition data was used to account for the loss of water to yield the estimate of p_{org}, the pure component vapor pressure, as $(3.0 \pm 0.1) \times 10^{-2}$ Pa.

RH	x_{org}	γ_{org}	da^2/dt ($m^2\ s^{-1}$)	p_{eff} / Pa	$[1+m_w/m_{org}]$	p_{org} / Pa
80	0.135	0.56	2.74E-15	4.12E-03	1.77	3.08E-02
70	0.205	0.71	4.51E-15	6.76E-03	1.47	3.17E-02
60	0.285	0.83	6.19E-15	9.29E-03	1.30	3.02E-02
50	0.37	0.92	7.98E-15	1.20E-02	1.20	2.92E-02
40	0.48	0.97	9.98E-15	1.50E-02	1.13	2.85E-02
30	0.6	0.99	1.25E-14	1.88E-02	1.08	2.93E-02
20	0.72	1.00	1.53E-14	2.30E-02	1.05	3.05E-02
11	0.85	1.00	1.79E-14	2.68E-02	1.02	3.09E-02
0	1	1.00	2.10E-14	3.15E-02	1.00	3.15E-02

Table S2: Table of calculations described in the main text for estimating the ratio of organic to AS in the organic-rich shell, under the assumption that the activity coefficients are unchanged in the shell with respect to the values in a binary system. x_{org}, $\frac{n_w}{n_{org}}$, x_{AS}, and $\frac{n_w}{n_{AS}}$ are derived from binary AIOMFAC calculations. $p_{org}{}'$ is the estimated vapor pressure of the organic assuming a binary shell composition and Δ is the ratio of $p_{org}{}'$ to the pure component vapor pressure P_{org} previously measured. $x_{org}(shell)$ is the required organic mole fraction in the shell to yield closure between $p_{org}{}'$ and p_{org} and the subsequent columns contribute to calculating the organic to AS ratio estimated to be present in the shell.

RH	x_{org}	n_w/n_{org}	x_{AS}(dis)	x_{AS}(undis)	n_w/n_{AS}	γ_{org}	da^2/dt (m^2 s^{-1})	p_{eff} / Pa	$[1+m_w/m_{org}]$	p_{org}' / Pa	Δ	x_{org}(shell)	n_{org}	$n_{w,org}$	n_{AS+w}	n_{AS}	org:AS
86	0.10	9.00	0.18	0.07	13.30	0.46	4.95E-16	7.43E-04	2.08	7.76E-03	-	-	-	-	-	-	-
76	0.17	4.88	0.27	0.11	8.11	0.64	1.60E-15	2.40E-03	1.59	1.39E-02	-	-	-	-	-	-	-
71	0.20	4.00	0.31	0.13	6.68	0.70	2.24E-15	3.36E-03	1.48	1.62E-02	-	-	-	-	-	-	-
65	0.24	3.17	0.36	0.16	5.24	0.77	3.93E-15	5.90E-03	1.38	2.31E-02	0.77	0.18	1.00	3.17	1.25	0.15	6.58
57	0.31	2.23	0.43	0.20	3.99	0.86	5.20E-15	7.81E-03	1.27	2.31E-02	0.77	0.24	1.00	2.23	0.96	0.14	7.27
47	0.41	1.44	0.51	0.26	2.85	0.93	6.90E-15	1.04E-02	1.17	2.31E-02	0.77	0.32	1.00	1.44	0.73	0.12	8.06
38	0.50	1.00	0.60	0.33	2.03	0.98	9.26E-15	1.39E-02	1.12	2.55E-02	0.85	0.42	1.00	1.00	0.36	0.07	14.13

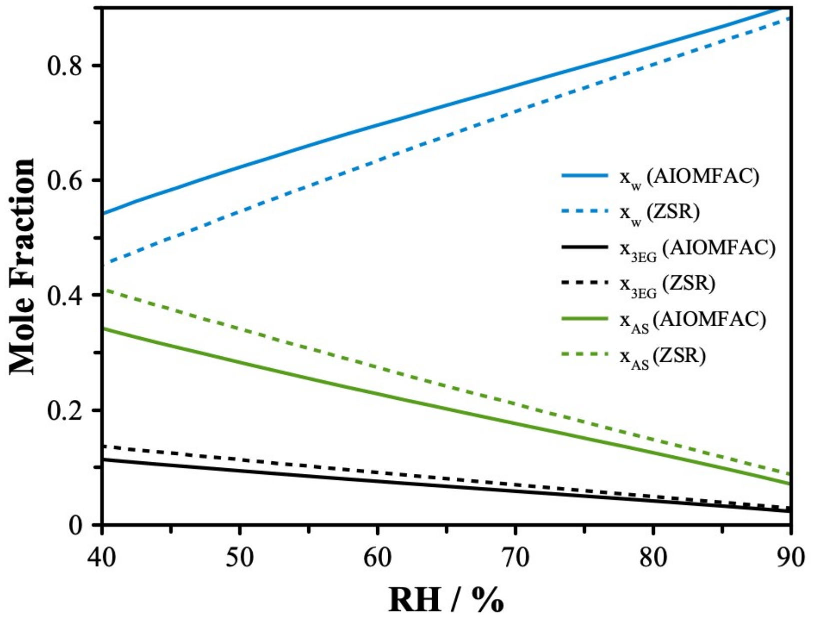

Figure S3: We use a ZSR assumption in this work to treat the composition of the ternary particle based on the AIOMFAC predictions of the binary systems. This facilitates calculating the variation in composition across an evaporation measurement. The ZSR assumption for the composition is compared here against a full AIOMFAC calculation of the ternary system. There is a systematic discrepancy on the order of 15% between the ZSR model and the AIOMFAC model for the equimolar mixture.

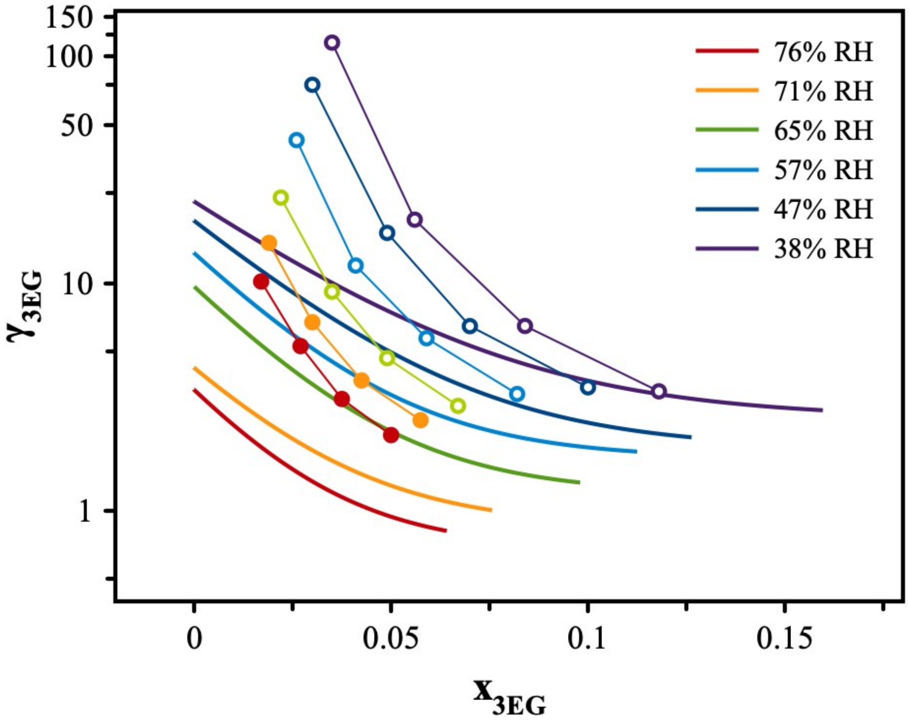

Figure S4: The activity coefficients of 3EG in a ternary mixture were found using the AIOMFAC model at each RH for different 3EG:AS ratios to span the range encountered experimentally during the evaporation process. The x-axis shows the mole fraction of 3EG in the evaporating mixture containing 3EG, AS and water, with the contribution of 3EG decreasing due to evaporation. For the AIOMFAC data, each point reflects a different calculation (with a different 3EG:AS ratio) and the mole fraction of 3EG in the particle is found assuming full dissociation of the AS. Solid points are shown to reflect predictions at RH above the LLPS RH, while the open circles are for predictions below the LLPS RH – it should be noted that LLPS formation is suppressed in the AIOMFAC model used for this comparison (forced single phase). The points are joined as a guide to the eye. The predictions are compared to the best-fit activity coefficients determined from the experimental evaporation data, shown as solid lines (without points).

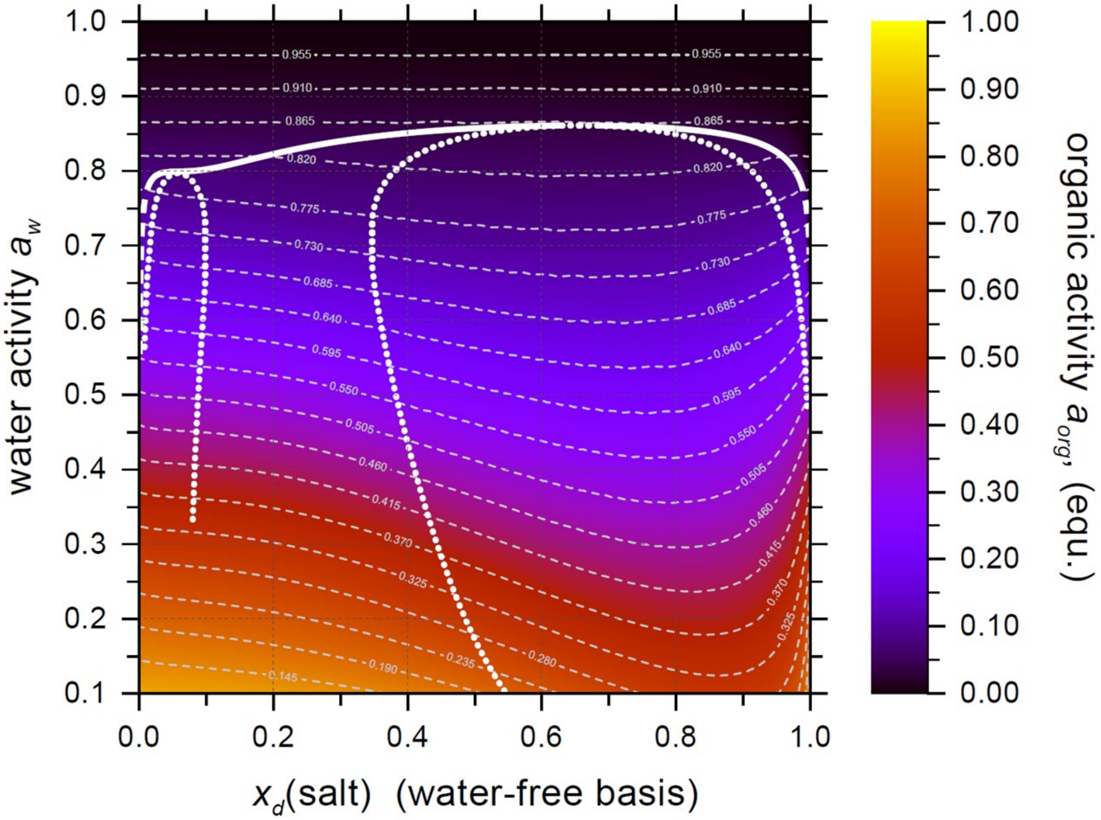

Figure S5: The AIOMFAC LLPS model was used to calculate the phase diagram of the ternary mixture of 3EG, AS and water at 293 K. The solid and dashed bold white line shows the binodal curve of LLPS (dashed in the range where dissolved AS is supersaturated with respect to solid AS, but kept in solution here), the dotted lines are the predicted spinodals (their interior phase space indicates unstable single-phase compositions). The color axis shows the organic activity of the equilibrium state (i.e. in the case of LLPS it is the organic activity in the two separate phases, equal in both). The thin dashed grey lines show contours of equilibrium state water activity (can be thought of as tie-lines in the LLPS region). The x-axis indicates the mole fraction of the salt (AS) in the dry mixture of solutes, indicative of the 3EG:AS ratio (hence showing composition on water-free basis).
